# Worcester Infirmary

**Published:** 1831

**Authors:** 


### WORCESTER INFIRMARY.

### RAPIDLY FATAL STRANGULATED HERNIA.

*Case.* John Hall, æt. 35, a healthy-looking labourer, admitted at 6, a.m. Sept. 14th, 1830, with very large strangulated scrotal hernia, very tense, and somewhat discoloured at the lower part. The tumour and the abdomen were very tender to the touch—pulse 100—slight hiccup—no vomiting since the previous evening. He had had a rupture for some time but never worn a truss ; it had descended at 5, p.m. on the preceding evening, whilst heaving a heavy ladder. He was seen by a surgeon at 11, p.m. and the taxis tried for two hours without effect. He had had one evacuation since the descent of the rupture.

At 10, a.m. the operation was performed by Mr. Pierpoint, the pulse being 150, the countenance very pale. On cutting through the integuments they were found to be gangrenous, and an immense quantity of intestine, of very dark colour, was found in the sac. The stricture was relieved, but the intestines could not be returned, and an incision was made into their lower part to evacuate their contents, and 1½ (qy. lb.) of grumous blood pressed out. The cut portion of intestine was secured by a ligature, and the integuments brought over it. There was much bloody fluid in the sac. The patient sank, and died at 2, p.m.

*Sectio Cadaveris.* The whole ileum was contained in the sac, and seemed as if tied by a ligature at the strictured part. The intestine was in a gangrenous state ; four yards and two inches of it were contained in the sac. The peritoneum and intestine in the abdomen were highly inflamed.

Can we wonder at the rapidity with which gangrene was induced in so large a mass of intestine, or, being induced, at the rapidity with which it proved fatal? In such a case the operation cannot be performed too early, the taxis cannot be employed too little.

### GUNSHOT-WOUND OF THE SCALP.

*Case.* Samuel Crook, æt. 27, was admitted August 18th, 1830, with severe gunshot wound of the head, situated over the left orbital process of the frontal bone, an inch in breadth, and two in length; the eye a good deal swollen and inflamed; a portion of the integuments and pericranium was carried away. The accident had been occasioned by a gun having gone off when he was drawing its charge. Leeches and a poultice were applied to the wound, the sloughs came away, and healthy granulations succeeded. On the 10th day there was shivering, succeeded by heat and sweating, vomiting, tongue loaded, brown and dry, and pain in the head. Blood was taken freely "from the head," and the patient purged with calomel and jalap. The wound suppurated, the symptoms grew worse, there came on paralysis of the right side, blindness of the right eye, tremors. On the 15th day from the occurrence of the accident, the trephine was applied over the denuded bone, and two circular pieces taken out, when half an ounce of fetid pus escaped, and a portion of dura mater was found to be sloughy. Immediately after the operation he moved his right arm and seemed to breathe more freely. He gradually sank, however, and died at 11, p. m.

*Sectio Cadaveris.* The whole of the left hemisphere of the brain was covered with pus, of which there was much also at the base, in the depression occupied by the middle lobe of the cerebrum; the pus extended down into the orbit, (which?) A portion of dura mater was sloughy, and adherent to the cerebrum to the extent of a shilling. The left hemisphere of the cerebrum was very much inflamed. No increased quantity of fluid in the ventricles.

We suppose from the context that the pus extended into the *left* orbit, but the case is loosely worded. The blindness was of the *right* eye, and, if the matter was in the other orbit, depended on the pus at the basis of the brain on the opposite side. It is rather singular that the presence of the matter in the left orbit produced no symptoms nor disturbance of the functions of that eye. Of late years, since the treatment of injuries of the head has been better understood and more scientifically practised: since, in fact, depletion has been generally employed in the early stages, the instances of secondary formation of matter on or under the dura mater have been comparatively rare. The present case adds another to the long list of failures from the use of the trephine for these secondary symptoms. From what we have seen of its employment in these cases, we should say that the unexperienced readers of Mr. Pott's works, would form a very erroneous opinion of its success. We have never seen it beneficial, although we are aware that it occasionally is so. Prevention by strict antiphlogistic regimen and depletion, is the grand point to be attended to.

